# Seroprevalence of *Neospora caninum-*specific antibodies in German breeding bitches

**DOI:** 10.1186/s13071-018-2683-1

**Published:** 2018-02-17

**Authors:** Rodolfo Villagra-Blanco, Lora Angelova, Theresa Conze, Gereon Schares, Andrea Bärwald, Anja Taubert, Carlos Hermosilla, Axel Wehrend

**Affiliations:** 1 Clinic for Obstetrics, Gynaecology and Andrology of Large and Small Animals with Veterinary Ambulance, Faculty of Veterinary Medicine, Justus Liebig University Giessen, 35392 Giessen, Germany; 2 Institute of Parasitology, Faculty of Veterinary Medicine, Justus Liebig University Giessen, 35392 Giessen, Germany; 3 Veterinarian Health Center Wiesbaden-Bierstadt, 65191 Wiesbaden, Germany; 4 Friedrich-Loeffler-Institut, Federal Research Institute for Animal Health, Institute of Epidemiology, 17493 Greifswald-Insel Riems, Germany

**Keywords:** *Neospora caninum*, Reproduction, Breeding bitches, Germany

## Abstract

**Background:**

*Neospora caninum* is an intracellular obligate apicomplexan parasite responsible for multisystemic lesions in dogs. Being definitive hosts and reservoirs, dogs excrete environmentally resistant oocysts. Breeding bitches represent a susceptible dog group and infected bitches may spread this parasite through transplacental transmission.

**Results:**

A total of 218 serum samples of German breeding bitches were collected to determine the presence of *N. caninum*. Antibodies were detected in 16 (7.33%) bitches using a commercial indirect enzyme-linked immunosorbent assay (ELISA). Immunoblotting analysis confirmed all seropositive samples detected by ELISA, proving that the animals were infected with *N. caninum*. The owners were interviewed regarding breed, age, environment, type, vaccine status, feeding habits and the presence of reproductive disorders. Seropositive animals were between the ages of two to seven years; three of them were kept in kennels while the others were household dogs, one of which was additionally a hunting dog. Owners of four seropositive bitches reported one gestation, while multiple pregnancies had been recorded for the other twelve bitches. Fourteen bitches were regularly vaccinated and six were fed with fresh raw meat.

**Conclusions:**

Although the results confirmed a low incidence of *N. caninum* seropositive German breeding bitches, further epidemiological and surveillance studies are required to complement our findings regarding the current situation of neosporosis in this specific canine population of Germany.

## Background

*Neospora caninum* is an apicomplexan obligate intracellular parasite that causes multisystemic lesions in dogs [1–5]. Dogs can act as definitive as well as intermediate hosts during *N. caninum* infections [6, 7]. Canine neosporosis is characterised by neuromuscular symptoms, such as ataxia, ascending paralysis, and other general nervous clinical signs [8]. Other manifestations include myocardial, pulmonary, dermatological, as well as reproductive disorders [3, 9–12]*. Neospora caninum* infections can occur through horizontal and vertical transmission of the parasite, i.e. a foetus may become infected transplacentally. In addition, dogs can be postnatally infected through the oral uptake of cysts from infected tissue material or sporulated *N. caninum* oocysts in contaminated food or water sources [11, 13, 14]. Oocysts are greatly significant in the spread and maintenance of this abortive agent, which is known to be highly tenacious [6, 7, 15].

Female dogs that have given birth to pups congenitally infected with *N. caninum* do not present any clinical signs [13]. Nevertheless, transmission of the protozoan to offspring in succeeding generations can occur [3, 16].

There are many diagnostic methods used to detect this parasite, such as histology, immunochemistry, serology, and conventional and real-time PCR [5, 17, 18]. Despite the fact that clinical canine neosporosis is rare, there are many reports on the seroprevalence of *N. caninum* in domestic and wild canines [10–13, 15]. Even among different canine populations with diverse roles and environments, distinct seroprevalences have been reported in stray [19], farm-rural [20–24], kennel [20] and urban dogs [21, 23–25].

European studies revealed differences in *N. caninum* seroprevalence; three of them were kept in kennels while the others were household dogs, one of which was additionally a hunting dog of various canine populations, presenting with15.3% seroprevalence in Denmark [9], 3.6% in Austria [26], 2.6–19.2% in Czech Republic [27], 17.2% in Serbia [28], 32.7% in Romania [29], 16.36% or 21.7% in Poland [25, 30], 10.9% in Italy [31], 12.2% in Spain [32],0.5% in Sweden [33] and 4% or 13% in Germany [34].

The aim of the present study was to determine the presence of *N. caninum* antibodies in German breeding female dogs and describe the characteristics of seropositive animals that may be correlated with this parasite and their potential involvement in reproductive disease.

## Methods

### Analysed population and sample size

Female dogs that showed optimal health parameters were presented for routine progesterone concentration measurements for ovulation determination at the Clinic for Obstetrics, Gynaecology and Andrology of the Justus Liebig University (JLU) Giessen, Germany. All bitches participating in this study were previously subjected to a clinical examination. A total of 218 samples were collected from March 2016 to June 2017 to determine the presence of *N. caninum* and the correlation between a current infection and reproductive disorders. Owners of seropositive animals were contacted and requested to complete a questionnaire that asked about breed, age, environment (indoors or outdoors, urban or rural), type of dog (farm, hunting, kennel, police, rescue, household/pet dogs), vaccination status (e.g. vaccinated against distemper virus, canine hepatitis virus, canine parvovirus, parainfluenza virus, *Leptospira* spp. and rabies), feeding habits, and reproductive disorders.

### Sample collection and additional information

Blood was collected by puncture of the cephalic vein. Then, the samples were transported at 5–10 °C. In the laboratory, samples were centrifuged for 5 min at 10000×*g*, and then sera were separated and frozen at -20 °C until further analysis.

### Enzyme-linked immunosorbent assay (ELISA)

The IDScreen® *Neospora caninum* Indirect Multi-species ELISA from IDVet® (Montpellier, France) was used for the detection of *N. caninum*-specific antibodies in canine serum samples. The same assay was employed in the studies by Sharma et al. [19], and Enăchescu et al. [35]. Sera were analysed, according to the ELISA-manufacturer’s instructions. For validation, positive control optical density (OD) averages and the difference between positive and negative control OD averages were evaluated. According to OD data of different serum samples, serum positive percentages (S/P) were calculated with respect to the average of the positive control sera using the following formula: S/P = (sample OD× 100) / (average OD of positive control). As recommended by the ELISA-manufacturer, samples that yielded S/P percentages of less than 40% were classified negative, samples with S/P values between 40–50% were weakly positive, and those with S/P values higher than 50% were assumed positive for *N. caninum* infection. The seropositive samples detected by ELISA and 10% of the remaining negative samples were further validated by immunoblotting assays.

### Immunoblot assays

Two immunoblot assays were performed: one immunoblot was based on total tachyzoite antigen (NC-1 strain of *N. caninum*; Dubey et al. [36] cultivated in MARC145 cells), while the second immunoblot relied on p38 tachyzoite antigen (NcSRS2) application after affinity purification, as previously described [37].

Total tachyzoite antigen immunoblot was performed as described previously [38] using 8 × 10^7^ tachyzoite pellets of *N. caninum* or purified NcSRS2 (p38, 0.05 μg per SDS-PAGE protocol) [37, 39]. Antigens were incubated in non-reducing sample buffer [2% (w/v) SDS, 10% (v/v) glycerol, 62 mM Tris-HCl, pH 6.8] for 1 min at 94°C, separated on12% SDS polyacrylamide minigels (60 × 70 × 1 mm), and transferred to PVDF membranes (Immobilon-P, Merck Chemicals GmbH, Darmstadt, Germany). After the transfer, membranes were blocked in PBS-TG consisting of PBS with 0.05% (v/v) Tween 20 (Sigma-Aldrich, Taufkirchen, Germany) and 2% (v/v) liquid fish gelatin (Serva, Heidelberg, Germany), cut into 50 strips, and examined as described below. To detect antibodies against *N. caninum* tachyzoite antigens, western blot membrane strips were incubated as previously described [38]. Dog sera were diluted 1:100 in PBS-TG, and then immunoreactions were detected using a peroxidase anti-dog IgG conjugate (Dianova, Hamburg, Germany) diluted 1:1000 in PBS-TG. Sera of naturally *N. caninum*-infected and non-infected dogs [40] were used as positive and negative control, respectively. In the case of total antigen detection, reactivity of the sera with non-reduced immunodominant *N. caninum* tachyzoite antigens (NC-IDA) of 17–19, 29, 30, 33, and 37 kDa Mr was examined. For purified NcSRS2, reactivity at 37–39 kDa was analysed [37].

## Results

**Fig. 1:**
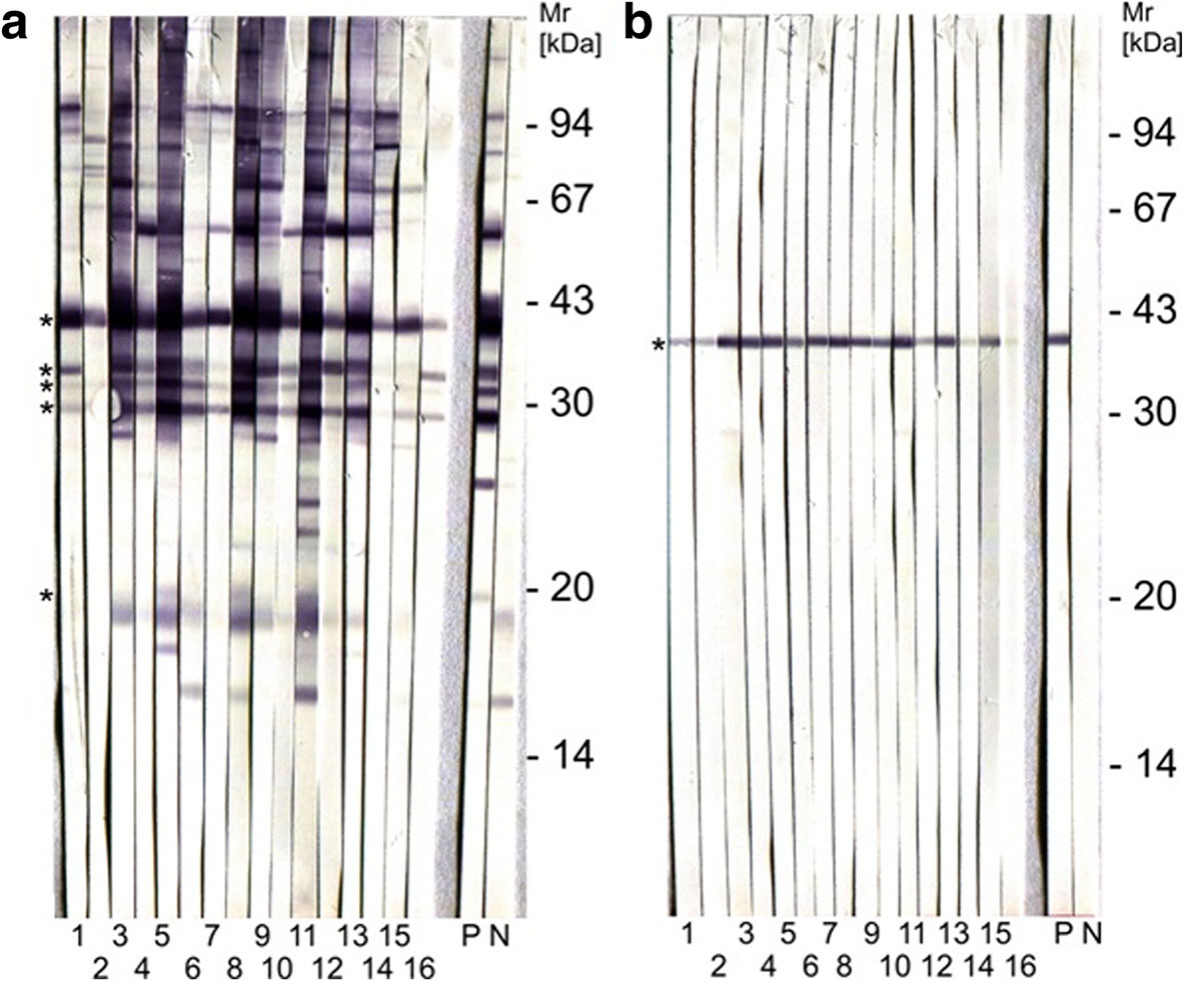
Immunoblot analysis of 16 *N. caninum*-seropositive German bitches. **a** Immunoblot with total tachyzoite antigen. Immunodominant antigens of 17–19, 29, 30, 33 and 37 kDa Mr are marked by asterisks. **b** Immunoblot using NcSRS2;the antigen is indicated with an asterisk. *Abbreviations*: P, positive control; N, negative control

Out of 218 analysed samples, 16 (7.33%) were positive for *N. caninum-*specific antibodies as determined by ELISA and reported S/P values higher than 50% (Table 1). Immunoblot-based analyses confirmed seropositivity of all samples detected positive by ELISA (Fig. 1) and the 20 representative samples found negative by ELISA (10%).

**Table 1:** Distribution of seronegative and seropositive sera of *N. caninum* according to the serum positive percentage (S/P) values determined with ELISA

Positivity percentage (SP)	*Neospora caninum* (%)
≤ 30 (negative serorreactors)	202 (92.6)
31–50 (low serorreactors)	0 (0)
51–70 (high positive serorreactors)	1 (0.6)
≥ 71 (very high positive serorreactors)	15 (6.8)
Total	218 (100.0)

Characteristics of the seropositive bitches are summarised in Table 2. The positive bitches were between two and seven years old, three (18.8%) were kept in kennels and the remaining were household animals (75%), including one (6.2%) that was also used for hunting. During sampling, four (25%) positive female dogs had been pregnant once, while the other 12 (75%) had had more than one birth as reported by the owners. Six (37.5%) seropositive individuals were fed with fresh raw meat not treated by cooking. Finally, 14 (87.5%) of the 16 seropositive bitches had a full vaccination program recorded.

**Table 2:** Characteristics of *N. caninum* seropositive breeding bitches

Animal	Breed	Age	Environment	Vaccination status	Feeding	Previous births
1	Rhodesian ridgeback	2	Household	*DHPPi*+LR	Raw meat	1
2	Shepherd dog	6	Household and kennel	*DHPPi* + LR	Dry food and raw meat	> 1
3	Giant schnauzer	6	Household	*DHPPi* + LR	Raw meat	> 1
4	Norwich terrier	7	Household	*DHPPi* + LR	Dry and wet food	> 1
5	Rhodesian ridgeback	5	Household	*DHPPi* + LR	Raw meat	> 1
6	German shepherd	4	Household	*DHPPi* + LR	na	> 1
7	Boston terrier	4	Household	*DHPPi* + LR	Dry food	> 1
8	English bulldog	-	Household	*DHPPi* + LR	Dry food	> 1
9	Giant schnauzer	6	Household	*DHPPi* + LR	Raw meat	> 1
10	Bernese mountain	6	Kennel	na	Dry food and raw meat	>1
11	English bulldog	-	Household	*DHPPi* + LR	Dry food	> 1
12	Bulldog	5	Household	*DHPPi* + LR	Dry food	> 1
13	Greater Swiss Mountain dog	4	Household	*DHPPi* + LR	Dry food	> 1
14	Rottweiler	7	Kennel	na	na	1
15	Unknown	4	Household	*DHPPi* + LR	Dry food	1
16	Austrian black and tan hound	2	Household and hunting	*DHPPi* + LR	Dry food	1

*Abbreviations*: *na* no answer, *DHPPi* + LR vaccination against distemper virus, canine hepatitis virus, canine parvovirus, parainfluenza virus, *Leptospira* spp. and rabies

## Discussion

The present study confirmed the presence of *N. caninum* antibodies in German breeding female dogs, which represent a susceptible *N. caninum*-infection dog group. Infected bitches may spread this parasite through transplacental transmission during successive pregnancies [41–43]. Immunoblot assays were used as a validation method for ELISA-positive and some ELISA-negative animals with the main purpose of avoiding false positive serological results and verifying the presence or absence of specific antibodies against *N. caninum* [25, 44].

The clinical and pathological isolation of *N. caninum* in an 11-week-old German puppy was previously reported [40]. Moreover, *N. caninum* faecal oocysts were found and cysts of this parasite were identified in German dogs [45, 46]. Previously, serological analyses of three German Doberman puppies from an infected bitch demonstrated the vertical transmission of *N. caninum* [42]. The low number of serologically positive dogs in this study (7.33%) is in agreement with previous seroprevalence obtained for German dogs with (13%) and without (4%) clinical signs of neosporosis [34] and in dogs from the German Federal State of Rhineland-Palatinate (4.45%) [47]. However, it should be noted that the novelty of this study relies on the low seroprevalence determined in canine breeding populations in Germany, specifically in the reproductive bitches population for which an *N. caninum* seroprevalence has not yet been described in the literature.

Transplacental transmission in dogs has been reported for experimental infections [48]; however, natural-neonatal canine neosporosis is rare and findings are variable, as not all litter puppies become seropositive [3]. Thus, frequent canine transplacental transmission is unlikely to occur in the absence of horizontal infection [3, 49], highlighting the importance of investigating additional canine horizontal infection routes of *N. caninum* in seropositive breeding bitches [3, 50]. All infection routes should be considered during the reproductive cycle of subclinical *Neospora*-infected bitches, especially considering that no drugs are known to prevent transplacental transmission [49]. Therefore, we also consulted with the owners of seropositive animals regarding risk factors of canine neosporosis identified in previous studies, such as breed [31], age [50], environment [51], type [20], vaccine status [52], and feeding habits [53].

Most of the individuals analysed were household, breeding female dogs. Several studies have demonstrated that European farm dogs have higher *N. caninum* seroprevalences than kennel, rescue, household, or urban dogs [20, 21, 54]. Seroprevalence was especially high in farm dogs that were kept with highly specialised dairy herds [22] or even with small ruminant flocks [23]; however, most of the studies mainly focused on this type of canine population, with only a few investigating household breeding dogs [3].

In the present study, four of the female dogs studied were found to have received raw meat as part of their diet. Horizontal transmission of *N. caninum* occurs through the intake of tissue cysts [55, 56]. Infection, as evidenced by shedding oocysts, was demonstrated in dogs after experimentally feeding them infected meat from goat and sheep [57].

Moreover, one positive household bitch was used for hunting proposes. Hunting dogs have an increased risk of being *N. caninum*-seropositive [24]. Possible contact with eviscerated infected wild animal carcasses (e.g. deer) might represent a potential source of infection [15, 29, 47]. In contrast with this observation, however, a serological study [9] found no statistical significance between seroprevalence of hunting and non-hunting dogs.

The vaccination status of the animals was also recorded to assure proper health status. In the present study, 14 out of 16 seropositive bitches were vaccinated. These data are in contrast with previous observations, in which vaccinated dogs had significantly lower seroprevalence compared with non-vaccinated canines [52]. The level of care provided by the dog owners regarding vaccination and diet were not correlated to *N. caninum* seropositivity.

Little is known about the clinical and economic consequences of canine neosporosis on the reproductive performance of breeding bitches and their progeny; therefore, further long-term studies are necessary to better understand the impact of neosporosis on breeding dog populations.

## Conclusions

We concluded that *N. caninum* infections exist in German breeding bitches at a very low prevalence. Nonetheless, further epidemiological studies are required to obtain more information regarding the seroprevalence of other German canine populations.
